# Accurate serology for SARS-CoV-2 and common human coronaviruses using a multiplex approach

**DOI:** 10.1080/22221751.2020.1813636

**Published:** 2020-09-08

**Authors:** Sophie van Tol, Ramona Mögling, Wentao Li, Gert-Jan Godeke, Arno Swart, Barbara Bergmans, Afke Brandenburg, Kristin Kremer, Jean-Luc Murk, Josine van Beek, Bas Wintermans, Johan Reimerink, Berend-Jan Bosch, Chantal Reusken

**Affiliations:** a Centre for Infectious Disease Control, National Institute for Public Health and the Environment (RIVM), Bilthoven, The Netherlands; b Virology Division, Department of Infectious Diseases and Immunology, Faculty of Veterinary Medicine, Utrecht University, Utrecht, The Netherlands; c Microvida, Elisabeth-Tweesteden Hospital, Tilburg, The Netherlands; d Izore Centre for Infectious Diseases Friesland, Leeuwarden, The Netherlands; e Department of Medical Microbiology and Immunology, Admiral De Ruyter Hospital, Goes, The Netherlands; f Department of Medical Microbiology, Bravis Hospital, Roosendaal, The Netherlands

**Keywords:** SARS-CoV-2, COVID-19, serology, immune profiling, micro-array

## Abstract

Serology is a crucial part of the public health response to the ongoing SARS-CoV-2 pandemic. Here, we describe the development, validation and clinical evaluation of a protein micro-array as a quantitative multiplex immunoassay that can identify S and N-directed SARS-CoV-2 IgG antibodies with high specificity and sensitivity and distinguish them from all currently circulating human coronaviruses. The method specificity was 100% for SARS-CoV-2 S1 and 96% for N antigen based on extensive syndromic (n=230 cases) and population panel (n=94) testing that also confirmed the high prevalence of seasonal human coronaviruses. To assess its potential role for both SARS-CoV-2 patient diagnostics and population studies, we evaluated a large heterogeneous COVID-19 cohort (n=330) and found an overall sensitivity of 89% (≥ 21 days post onset symptoms (dps)), ranging from 86% to 96% depending on severity of disease. For a subset of these patients longitudinal samples were provided up to 56 dps. Mild cases showed absent or delayed, and lower SARS-CoV-2 antibody responses. Overall, we present the development and extensive clinical validation of a multiplex coronavirus serological assay for syndromic testing, to answer research questions regarding to antibody responses, to support SARS-CoV-2 diagnostics and to evaluate epidemiological developments efficiently and with high-throughput.

## Introduction

Severe acute respiratory syndrome coronavirus 2 (SARS-CoV-2) emerged late 2019 and has since then spread globally with more than 13.5 million infections and 580,000 fatalities as at 17 July 2020 [1]. Currently, there is no approved medication or vaccine available for coronavirus disease 2019 (COVID-19). Control measures consist of a combination of physical distancing, self-isolation of symptomatic individuals, isolation of confirmed cases and tracing and quarantining of their contacts [2,3].

To diagnose a SARS-CoV-2 infection, reverse transcription polymerase chain reaction (RT-PCR) on upper respiratory tract samples is the recommended method [4,5]. However, serology is occasionally imperative to complement RT-PCR findings as a lack of clinical sensitivity is observed for RT-PCR-based diagnostics in patients with a strong clinical suspicion for COVID-19 [6,7]. More importantly, serology is crucial for SARS-CoV-2 epidemiology and public health research as it enables assessment of the presence of SARS-CoV-2 infection in putative animal reservoirs and of SARS-CoV-2 prevalence in different human (sub)populations in time, thereby providing insight in levels of possible protective immunity and the true mortality rates including the proportion of asymptomatic and mild cases.

SARS-CoV-2 expresses four major structural proteins, i.e. the spike (S), envelop (E), membrane (M) and nucleocapsid (N) proteins [8]. SARS-CoV-2 induces an antigen-specific antibody response with S and N considered to have the highest immunogenicity [9] while the sensitivity, specificity and functionality of antibodies elicited against these antigens remain to be characterized . The S-protein interacts with angiotensin-converting enzyme 2 (ACE2) which mediates host cell entry of the virus. The N-terminal S1 subunit comprises the ACE2 receptor-binding domain (RBD) while the C-terminal S2 subunit is responsible for virus-cell membrane fusion. The N protein packages the viral genome into helical virions and has a role in subgenomic RNA transcription and genome replication [8,10,11]. Recently, many serology-based diagnostic tools have come on the market, mainly enzyme-linked immunosorbent assays (ELISAs) and lateral flow assays (LFAs) based on the S and/or N antigens. These commercial tests have shown a high variability in test performance and are all single-plex assays [12,13]. The choice of serology platform strongly depends on the intended use (e.g. individual patient diagnostics vs (sub) population serology) and the associated minimum requirements for sensitivity and specificity.

Here, we describe the development, validation and clinical evaluation of a protein micro-array (PMA) as a quantitative multiplex immunoassay that can identify S and N-directed SARS-CoV-2 antibodies with high specificity and sensitivity. It enables distinct detection of SARS-CoV-2 antibodies from all five currently circulating human coronaviruses (HCoVs). Four HCoVs, i.e. HCoV-OC43, HCoV-HKU1, HCoV-NL63 and HCoV-229E, follow a seasonal transmission pattern and are associated with mild respiratory symptoms. A fifth HCoV virus, MERS-CoV has a limited circulation in the human population upon sporadic spill-over from its dromedary reservoir and is associated with severe illness [14,15]. The multiplex approach allows for antibody profiling, which potentially provides an increased insight in immune responses towards all currently circulating human-infecting coronaviruses. The assay was evaluated in various cohorts of COVID-19 cases of different disease severities and proved to be suitable for population-based SARS-CoV-2 immune response studies.

## Methods

### Study cohorts

The specificity of the HCoV protein micro-array (HCoV-PMA) was assessed with the following anonymized cohorts (supplementary table S1): (a) healthy blood donors, age 18–79 years, 2016 (*n* = 74); (b) acute *cytomegalovirus* (CMV) patients, 2016 (*n* = 10); (c) acute *Epstein–Barr virus* (EBV) patients, 2016 (*n* = 10); (d) patients with a 2 months earlier PCR-confirmed common coronavirus infection HCoV-229E (*n* = 23), HCoV-NL63 (*n* = 19), HCoV-HKU1 (*n* = 6) or HCoV-OC43 (*n* = 32), age >60 years, 2011–2015 [16,17]; (d) patients with PCR-confirmed non-CoV respiratory infections, e.g. *Influenza A virus, Influenza B virus, human metapneumovirus* and *rhinoviru*s, age >60 years, 2011–2015 (*n* = 100) [16,17]; (e) patients with respiratory complaints of unknown aetiology, age <18 years, 2019 (*n* = 50). The sensitivity of the HCoV-PMA was assessed with an anonymized cohort of RT-PCR-confirmed Dutch COVID-19 cases, age 2–91 years, 2020 (*n* = 449 sera for *n* = 330 cases). Age, gender, disease severity and timing of sample collection in days post onset of illness were known for 296 cases (Table S2).

Sera from common CoV cases and non-CoV respiratory cases were obtained from a previous study at the National Institute of Public Health and the Environment (METC Noord-Holland, http://www.trialregister.nl; NTR3386 and 4818 [16,17]). The current study was performed in accordance with the guidelines for sharing of patient data of observational scientific research in emergency situations as issued by the Commission on Codes of Conduct of the Federation of Dutch Medical Scientific Societies (https://www.federa.org/federa-english).

### Protein expression

The antigens HCoV-229E S1 (GenBank JX503061.1), HCoV-HKU1 S1 (ADN03339.1), HCoV-OC43 S1 (AIX10763), HCoV-NL63 S1 (ABE97130.1) and MERS-CoV S1 (KJ650297.1) were produced in-house as recombinant proteins in a stably transfected mammalian HEK-293 cell-line [18]. pcDNA3.1 (Invitrogen, Carlsbad CA, USA) plasmids encoding the S1 protein directly followed by a rabbit IgG-Fc-tail and His-tag were transfected into HEK293 cells. Secreted recombinant protein was purified from culture supernatant by fast protein liquid chromatography (FPLC) according to the manufacturer’s instructions, by using HisPur Ni-NTA chromatography columns (ThermoFisher Scientific Inc, Breda, the Netherlands). Purified protein was concentrated with 10 kDa Amicon centrifugal filter units (Millipore, Zwijndrecht, the Netherlands) and stored at −80°C until further use. Expression of the correct protein was verified by sequencing of the incorporated insert in the genome of the transfected cells. Spike protein S1 (QHD43416.1) of SARS-CoV-2 followed by a human IgG-Fc-tail was recombinantly expressed by transfection of mammalian 293F cells and affinity purified using Protein-A Sepharose beads (catalogue no. 17-0780-01; GE Healthcare) as described by Okba and colleagues [19]. The nucleocapsid protein N of SARS-CoV-2 was obtained from a commercial source (Sino Biological, Eschborn, Germany; Cat: 40588-V08B).

### Preparation of human coronavirus protein micro-array

HCoV-PMA slides were essentially produced as described previously [20]. Antigens (HCoV-OC43, HCoV-HKU1, HCoV-229E, HCoV-NL63, MERS-CoV S1 at 0.75 mg/ml; SARS-CoV-2 S1 at 0.65 mg/ml; SARS-CoV-2 N at 0.40 mg/ml) were spotted in duplicate in three drops of 333 pL each on 24-pads nitrocellulose-coated slides (ONCYTE AVID, GraceBio Labs, Bend, USA) by using a non-contact Marathon Arrayjet micro-array spotter (Roslin, UK). Printed micro-array slides were pre-treated with Blotto blocking buffer (ThermoFisher) to avoid non-specific binding as previously described [14]. Sera were tested in four 4-fold dilutions starting at 1:20, diluted in Blotto buffer containing 0.1% Surfact-Amps20 (ThermoFischer) as previously described [14,20]. Subsequently, slides were incubated with goat anti-human IgG, F(ab’)2 fragment specific, Alexa Fluor 647-conjugated (Jackson Immuno Research, West Grove, USA), diluted 1:1000 in Blotto buffer with 0.1% Surfact-Amps20 as described. Incubation steps were followed by a washing step with 1× phosphate-buffered saline with 0.1% Tween. After the last wash, slides were washed with sterile water and dried. Day-to-day variations were monitored by including a SARS-CoV-2 positive control serum in each test round. An in-house SARS-CoV-2 standard was included in each test batch to correct for test-to-test variation. If the titre of the positive control deviated more than two-fold from the expected titre, the test batch was rejected and repeated.

### Data analysis

Data analysis was performed as previously described [20]. Briefly, ScanArray Express software version 4.0.0.0004 (PerkinElmer, Waltham, USA) was used to quantify fluorescent signals. Maximum signal readout was fixed at 65,535 and minimal signal readout at 3000 fluorescent units. The mean of the median spot fluorescence of duplo measurements was plotted into dose–response curves per antigen for each serum using R studio v4.0.0, package “DRC” version 2.3-7 (R studio, Boston, USA). A representative theoretical antibody titre (EC50) was chosen at the 50% response on the dose–response curve, analogous to the median infectious dose (ID50) in the dose–response theory. If the median fluorescence measurements of the used serum dilutions were outside the linear range of the sigmoidal dose response curves, no titre could be calculated. Therefore, if the mean fluorescence signal was below 70% of the maximum signal for the lowest dilution (<45.000), no titre was calculated and a value of 10 was ascribed for further analysis. Similarly, if the mean fluorescence signal was above 30% of maximum signal for the highest dilution (>20.000), the titre was ≥1280 and was ascribed the value of 1280 for further analysis. Heatmaps were generated using the “heatmap.2” function from the “gplots” package in R v3.6.0 (R studio). Micro-array titres were log10-transformed with the lowest values set at 1 (green) and highest values set at 3 (red). The dendrogram was calculated by using hierarchical clustering using complete linkage [21]. The trend in antibody dynamics in Figure 3 was visualized using a smoothed interpolating Loess curve [22] that was overlaid for each severity of disease and antigen, using default settings of a span of 0.75 and degree 2.

### SARS-CoV-2 virus neutralization tests (VNT_50_)

SARS-CoV-2 virus neutralization tests were performed exactly as described [23]. Two-fold dilutions (starting at 1:10) of heat-inactivated sera (30 min. 56°C) were incubated with 100 TCID_50_ of SARS-CoV-2 strain HCoV-19/Netherlands/ZuidHolland_10004/2020 (EVAg cat.nr. 014V-03968) at 35°C, 5% CO_2_ for 1 h in 96-wells plates. African green monkey (Vero-E6) cells were added in a concentration of 2 × 10^4^ cells per well and incubated for three days at 35°C in an incubator with 5% CO_2_. The serum virus neutralization titre (VNT_50_) was defined as the reciprocal value of the sample dilution that showed a 50% protection of virus growth. Samples with titres ≥10 were defined as SARS-CoV-2 seropositive.

## Results

### Specificity

**Figure 1.**
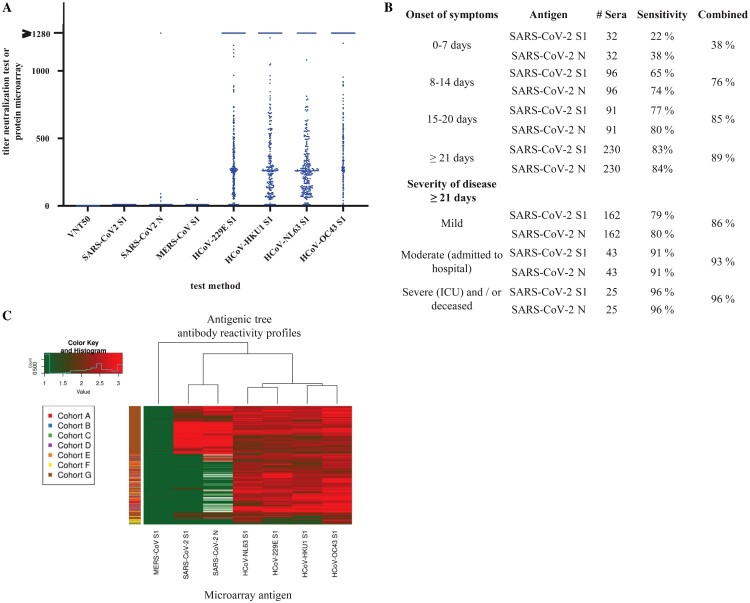
Validation of SARS-CoV-2 protein micro-array. (A) Specificity of SARS-CoV-2 VNT_50_ and SARS-CoV-2 S1 and N antigens on the HCoV-PMA measured with pre-COVID-19 sera from different cohorts that were sampled between 2011 and 2019. (B) Sensitivity of SARS-CoV-2 S1 and N antigen protein micro-array determined with 449 sera of 330 RT-PCR-confirmed SARS-CoV-2 cases, analysed by days post onset symptoms and severity of disease: i.e. mild (no admission to hospital), moderate (admission to hospital, but not ICU) and severe (admitted to ICU and/or deceased). (C) Heatmap displaying log10-transformed micro-array titres of 255 pre-COVID-19 cohorts sera, and sera of 449 RT-PCR-confirmed SARS-CoV-2 cases against antigens of all currently circulating coronaviruses (colour key of titres indicated in the top left corner: green – negative/low titres, red – high titres, white – not done). The cohorts are: (A) healthy blood donors, (B) acute cytomegalovirus patients, (C) acute Epstein–Barr virus patients, (D) patients with recent PCR-confirmed seasonal HCoV infection, (E) patients with recent non-coronavirus influenza-like-illness infection, (F) patients with respiratory complaints of unknown aetiology and (G) cases with RT-PCR-confirmed SARS-CoV-2 infection. All sera from cohorts A-F were sampled between 2011 and 2019.

The specificity of the HCoV-PMA for detection of SARS-CoV-2 specific IgG was assessed using a panel (n = 324 for SARS-CoV-2 S1, *n* = 162 for SARS-CoV-2 N) consisting of pre-COVID-19 outbreak sera from healthy donors, sera from patients with (un)diagnosed respiratory complaints and sera of patients with a confirmed seasonal HCoV infection (table S1, Figure 1(A)). None of these sera had an IgG titre against SARS-CoV-2 S1 (specificity 100%). Six sera, i.e. of two healthy donors, two recent HCoV-229E and two acute CMV patients, were reactive against SARS-CoV-2 N (specificity 96%). One HCoV-OC43 serum gave a titre with MERS-CoV S1. For comparison, none of the 324 sera were able to neutralize SARS-CoV-2 in the VNT_50_. The expected high prevalence of antibodies against seasonally circulating HCoV in the general population [24–26] was reflected in the extensive reactivity of all sera against the seasonal HCoVs S1 antigens, i.e. 93% for HCoV-229E, 94% for HCoV-HKU1, 97% for HCoV-NL63 and 99% for HCoV-OC43 (Figure 1(A)).

### Sensitivity

The clinical sensitivity of the HCoV-PMA for detection of SARS-CoV-2 specific IgG was assessed, using a panel of 449 sera of 330 RT-PCR-confirmed COVID-19 cases (Figure 1(B)). The overall combined sensitivity for samples collected ≥21 days post onset symptoms (dps) (*n* = 230) was 89%, but 83% and 84% for the single antigens S1 and N respectively. In samples collected in the third week of illness (*n* = 91) the combined sensitivity was 85% while the single antigen sensitivities were 77% (S1) and 80% (*N*). In the first and second week upon the onset of illness, the combined sensitivities were respectively 38% and 76%.

A breakdown by disease severity in the sample set taken ≥21 dps demonstrated an increase in sensitivity with increasing disease severity (Figure 1(B)) with sensitivities of 86% (mild), 93% (moderate) and 96% (severe).

An important feature of a multiplex CoV serology tool is its capacity to differentiate antibody responses at virus-specific level. To confirm the overall correct grouping in our assay of the serum cohorts according to their known exposure history, we visualized IgG binding to all seven antigens for the 449 sera of the sensitivity cohort and 255 sera in the specificity cohort into a heatmap (Figure 1(C)). Sixty-nine sera of the 324 sera in the specificity cohort that did not give an initial response titre for SARS-CoV-2 S1 and N on the HCoV-PMA were not titrated further for the seasonal CoVs due to limited availability of the sera and were omitted from the heatmaps. The heatmaps clearly yielded the expected differentiated clusters in reactivity for the seasonal *Alphacoronaviruses* (HCoV-NL63 and HCoV-229E), seasonal *Betacoronaviruses* (HCoV-OC43 and HCoV-HKU1), and the emerging *Betacoronaviruses* MERS and SARS-CoV-2 thereby confirming the value for distinctive serology of this multiplex approach.

### Correlation with functional antibodies

**Figure 2.**
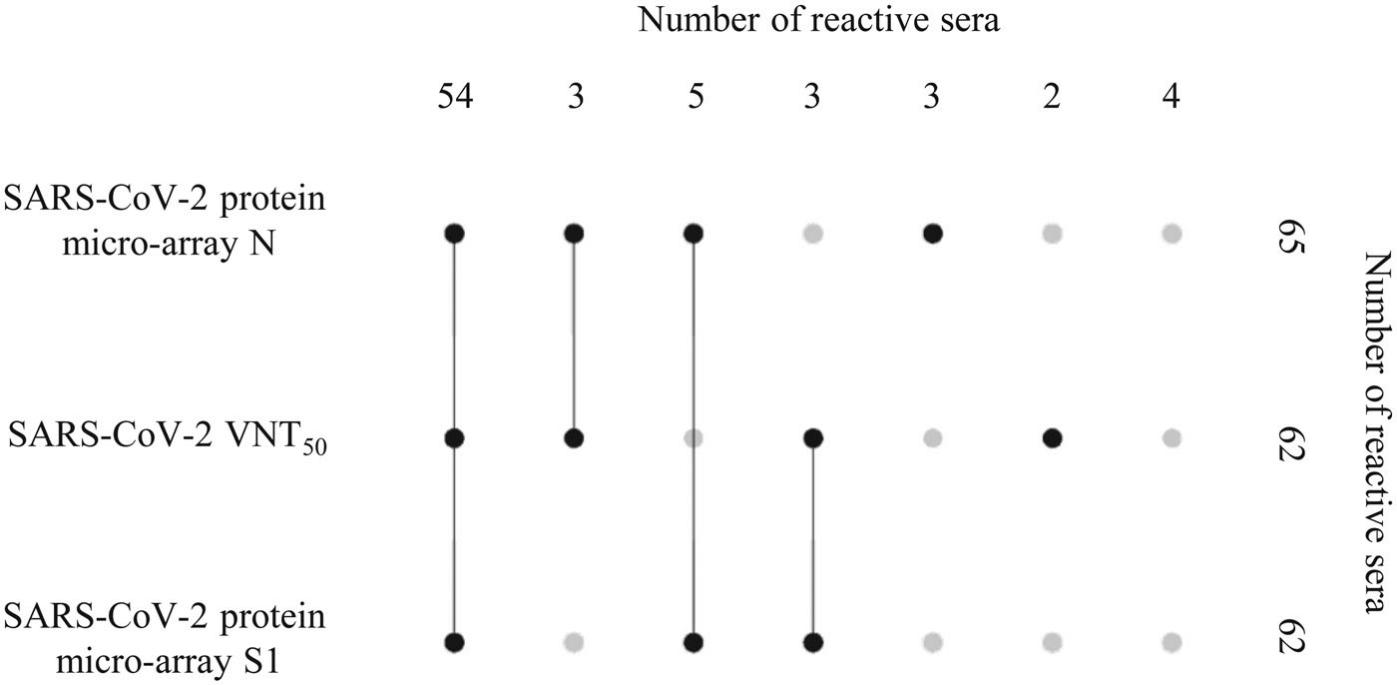
UpSet plot [40] visualizing the relationships between data sets obtained with three different assays, i.e. SARS-CoV-2 micro-array S1, SARS-CoV-2 micro-array N, SARS-CoV-2 VNT_50_, and 74 sera from RT-PCR-confirmed SARS-CoV-2 cases that were sampled ≥21 days after onset of symptoms. Top row depicts the number of 74 serum samples that could be detected (plotted black dots) or not (plotted grey dots) in different combinations of the three assays. Column on the right side depicts how many sera of the total number of 74 sera that could be identified (black dots) or not (grey dots) using the respective individual test methods.

To assess the correlation between the detection of SARS-CoV-2 IgG titres with the HCoV-PMA and the presence of protective antibodies, we tested a random subset of 74 serum samples collected ≥21 dps of PCR-confirmed COVID-19 cases in the VNT50 (Table S2, Figure 2). Sixty-two samples showed a SARS-CoV-2 IgG titre with the S1 antigen and 65 samples with the N antigen. Fifty-seven of the 62 S1-reactive samples (92%) were confirmed to have SARS-CoV-2 neutralizing antibodies (Figure 2). For hospitalized cases (*n* = 35) there was a 100% correlation between IgG reactivity for S1 and the presence of neutralizing antibodies whereas only 81% of S1-reactive sera from non-hospitalized, mild cases (*n* = 27) showed neutralizing potency (data not shown). Eighty-eight percent (57 of 65) of the sera with an IgG titre against N were positive in the VNT_50_ (Figure 2). The correlation was 100% for hospitalized cases (*n* = 35) and 73% for mild cases (*n* = 30) (data not shown). Likewise, 60 of 62 sera (97%) with neutralizing antibodies were positive for one or both SARS-CoV-2 antigens on the HCoV-PMA (Figure 2). Notably, the median titres of the sera that were reactive in all three methods (*n* = 54) were significantly (*p* < 0.0001, two-tailed Mann–Whitney *U*-test) higher than the titres of the sera that were reactive in one or two methods (*n* = 16), i.e. for VNT_50_ 240 vs 5.5, for PMA-S1 1280 vs 24 and for PMA-N 1280 vs 48.

### SARS-CoV-2 antibody kinetics and clinical manifestation

**Figure 3.**
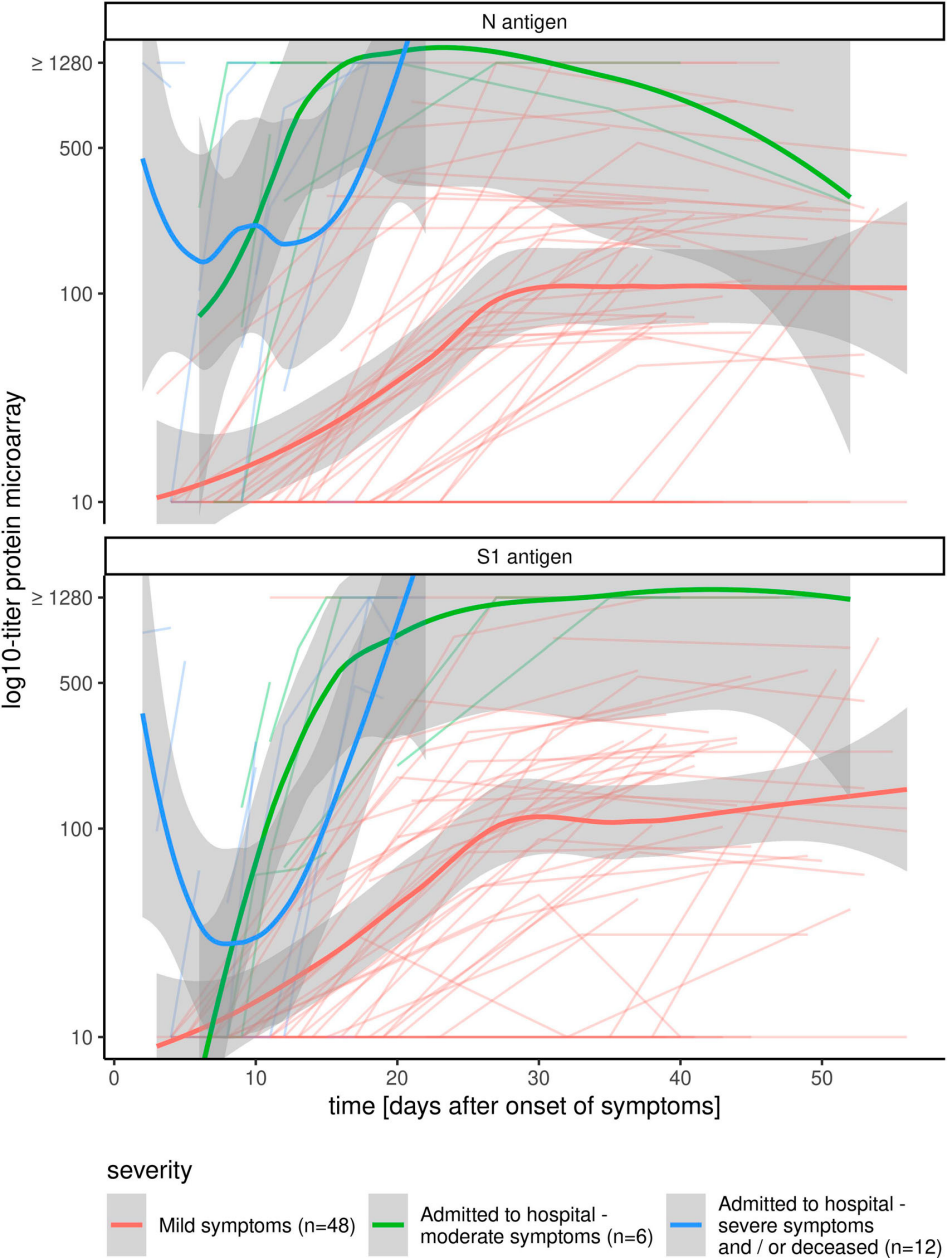
SARS-CoV-2 antibody kinetics and clinical manifestation. Longitudinal micro-array titres of individual cases with varying degrees of severity of COVID-19 infection: cases with mild symptoms (red, *n* = 47), patients with moderate symptoms that were admitted to the hospital, but not in intensive care unit (green, *n* = 6), patients with severe symptoms that were admitted to the intensive care unit of the hospital and/or died to COVID-19 infection (blue, *n* = 12). Bold lines depict the fitted curves with the 95% confidence interval for the three disease categories.

Finally, we applied the HCoV-PMA for a longitudinal assessment of SARS-CoV-2 response titres in individual cases with varying degrees of COVID-19 disease severity. Hospitalized patients, both in common COVID-19 wards (moderate, *n* = 6) and admitted to the intensive care unit (ICU) and/or deceased (severe, *n* = 12), demonstrated a more rapid and higher level antibody response to S1 and N than non-hospitalized infected persons (*n* = 47) with in the latter group an absent seroconversion for 10 patients as measured up to 39–56 dps with the S1 antigen (Figure 3). For mild cases a median highest titre of 245 (IQR 103–506) for S1 was observed and of 235 (IQR 107–335) for N. The highest titres were measured at a median of 39 dps (IQR 35–44) and 37 dps (IQR 31–43) against S1 and N respectively. For moderately ill patients the median titres were 1280 (IQR 398–1280) for S1 and 1280 (IQR 1105–1280) for N with the first day of highest titre at 16 dps (IQR 14–29) for S1 and 14 dps (IQR 10–22) for N. For ICU-admitted and/or deceased patients the highest titres were measured for the first time with a median of 10 dps (IQR 5.5–18) for S1 and 11 dps (IQR 5.3–17) for N. Median highest titres were 637 (IQR 229–1280) for S1 and 1280 (IQR 987–1280) for N (Figure 3, data not shown).

## Discussion

We presented a multiplex, quantitative approach for the specific and sensitive detection of IgG to all six currently circulating HCoVs, including both seasonal, zoonotic and pandemic HCoVs. The assay was built upon our previously established and successfully applied platform for comparative serology for emerging coronaviruses in human and veterinary samples [14,15,27]. Although SARS-CoV-2 is con-specific with SARS-CoV, SARS-CoV circulation in the human population has been eliminated over 15 years ago [8,28]. Therefore, antigens representing this virus were not included in the HCoV-PMA. The multiplex-serology tool can be used in epidemiological studies to estimate the infection burden of SARS-CoV-2 in different cohorts, as a research tool to profile HCoV antibody responses in relation to clinical outcomes and functionality of antibody responses, and as a diagnostic tool. In contrast to conventional ELISAs, the technique requires only minimal amounts of antigen and sample, i.e. dried blot spots or fingerstick blood which is of high logistic value in large population studies [29]. It allows for simultaneous high-throughput testing against 100 different antigens. To increase the applicability of the tool in non-specialized laboratories, an alternative, low-cost visualization (staining) strategy will be implemented that enables the use of field scanners and roll-out of the methodology to non-reference laboratories.

Population studies in low seroprevalence settings, e.g. European countries (<10% [30]), require an assay with high specificity to ensure an acceptable predictive value of positive test outcomes [31]. Our test showed a specificity of 100% for SARS-CoV-2 S1 and 96% for N antigen based on an extensive syndromic (*n* = 230) and population panel (*n* = 94), both panels were expected and confirmed to have a high seroprevalence (≥93%) for all seasonal HCoV [24–26].

To gain insight into the application of the HCoV-PMA for both patient diagnostics and population studies, we assessed the sensitivity of the tool in a clinically heterogeneous cohort of confirmed COVID-19 cases, hence reducing the overall sensitivity of the assay versus studies where only severe patients were included. As it was becoming evident from international studies that antibody responses should be assessed preferably at least 3 weeks dps [13,32,33], we analysed the assay sensitivity in different episodes of sampling. Indeed the highest sensitivity, 96%, was achieved in samples taken ≥21 dps from patients with the highest disease severity. The combined sensitivities we observed for the four different periods of sampling were in line with the observations in the Cochrane assessment of 54 serology studies by Deeks and colleagues [13]. They observed IgG sensitivities of 29.7% (95% CI 22.1–38.6), 66.5% (95% CI 57.9–74.2), 88.2% 95% CI (83.5–91.8), 80.3% (95%CI 72.4–86.4) for respectively 1–7 dps, 8–14 dps, 15–21 dps and 22–25 dps. However, cautiousness is indicated when comparing the diagnostic performance of different types of assays. Besides the timing of sampling, sensitivity data should be interpreted in the context of type of antigen used and severity of disease. A combined sensitivity of 86% in mild cases and the observed 100% specificity make the HCoV-MPA a powerful tool for cohort and population studies. Options to improve the assay sensitivity by employing different antigen configurations, e.g. use of S-trimers [34] are currently being explored.

Similar to other studies [19,32,33,35] we observed an absent or delayed, and lower antibody response to SARS-CoV-2 S1 and N in cases with mild disease, which likely leads to an underestimation of the infection burden of SARS-CoV-2 in population seroprevalence studies. A main focus of population seroprevalence studies is to assess the protective immunity status in (sub)populations to inform risk management. Insight in the correlation between antibody measurements with the applied screening tool and the presence of protective antibodies is needed. In hospitalized patients, we observed a 100% correlation with the presence of neutralizing antibodies for HCoV-PMA reactivity against S1 and N antigens. In mild cases this was only 81% and 73% respectively, clearly indicating a pitfall of protective immunity assessment in the general population based on antibody measurements with the current assay. However, protective immunity is multifactorial and community level immunity should not be addressed based on antibody level assessments only [36].

Besides a highly reliable distinction of SARS-CoV-2 immune responses from responses against the four seasonal HCoV, the HCoV-PMA provides insight into IgG responses against these common cold, seasonal viruses [14]. Our data confirmed the high overall prevalence (≥93%) of immune responses against these viruses in the general population. Further studies using the HCoV-PMA will investigate amongst others correlations between the level of pre-existing immunity to seasonal HCoVs and disease outcome for COVID-19.

As illustrated here, the strength of a multiplex-serology approach is that combining measured responses to multiple antigens of a specific pathogen can improve the sensitivity of the assay [37,38]. Furthermore, multiplex serology will offer an elegant approach to syndromic testing, enabling the simultaneous assessment of (recent) infections with multiple respiratory pathogens, e.g. emerging and seasonal HCoVs, influenza A and B viruses and RSV [14,20,39]. To increase the diagnostic value of the HCoV-PMA, the tool will be validated for the determination of IgM and IgA responses. In addition, the performance and applicability of the assay will be evaluated further in large cohort studies.

In conclusion, we present the development and clinical validation of a quantitative multiplex HCoV serology assay that can detect and distinguish SARS-CoV-2 antibodies with high sensitivity from antibodies against circulating HCoV and other respiratory viral pathogens. It provides a valuable high-throughput tool for HCoV immune profiling at (sub)population level and to support patient diagnostics where RT-PCR lacks in sensitivity.

## Supplementary Material

Table_S2.xlsx

Table_S1.xlsx
